# Assessing temporal associations between environmental factors and malaria morbidity at varying transmission settings in Uganda

**DOI:** 10.1186/s12936-016-1549-2

**Published:** 2016-10-19

**Authors:** Ruth Kigozi, Kate Zinszer, Arthur Mpimbaza, Asadu Sserwanga, Simon P. Kigozi, Moses Kamya

**Affiliations:** 1 Uganda Malaria Surveillance Project, Kampala, Uganda; 2 Children’s Hospital Informatics Program, Boston Children’s Hospital, Boston, MA USA; 3 College of Health Sciences, Makerere University, Kampala, Uganda

**Keywords:** Malaria, Uganda, Cross-correlations, Early warning system, Environment

## Abstract

**Background:**

Environmental factors play a major role in transmission of malaria given their relationship to both the development and survival of the mosquito and parasite. The associations between environmental factors and malaria can be used to inform the development of early warning systems for increases in malaria burden. The objective of this study was to assess temporal relationships between rainfall, temperature and vegetation with malaria morbidity across three different transmission settings in Uganda.

**Methods:**

Temporal relationships between environmental factors (weekly total rainfall, mean day time temperature and enhanced vegetation index series) and malaria morbidity (weekly malaria case count data and test positivity rate series) over the period January 2010–May 2013 in three sites located in varying malaria transmission settings in Uganda was explored using cross-correlation with pre-whitening. Sites included Kamwezi (low transmission), Kasambya (moderate transmission) and Nagongera (high transmission).

**Results:**

Nagongera received the most rain (30.6 mm) and experienced, on average, the highest daytime temperatures (29.8 °C) per week. In the study period, weekly TPR and number of malaria cases were highest at Kasambya and lowest at Kamwezi. The largest cross-correlation coefficients between environmental factors and malaria morbidity for each site was 0.27 for Kamwezi (rainfall and cases), 0.21 for Kasambya (vegetation and TPR), and −0.27 for Nagongera (daytime temperature and TPR). Temporal associations between environmental factors (rainfall, temperature and vegetation) with malaria morbidity (number of malaria cases and TPR) varied by transmission setting. Longer time lags were observed at Kamwezi and Kasambya compared to Nagongera in the relationship between rainfall and number of malaria cases. Comparable time lags were observed at Kasambya and Nagongera in the relationship between temperature and malaria morbidity. Temporal analysis of vegetation with malaria morbidity revealed longer lags at Kasambya compared to those observed at the other two sites.

**Conclusions:**

This study showed that temporal associations between environmental factors with malaria morbidity vary by transmission setting in Uganda. This suggests the need to incorporate local transmission differences when developing malaria early warning systems that have environmental predictors in Uganda. This will result in development of more accurate early warning systems, which are a prerequisite for effective malaria control in such a setting.

## Background

Malaria is a major public health challenge worldwide with 3.3 billion people at risk of infection, resulting in over 214 million cases and an estimated 438,000 deaths in 2015 [1]. In Uganda, it is the leading cause of morbidity and mortality accounting for 40 % of the hospital outpatient visits, 20 % of hospital admissions, and 14 % of hospital deaths [2]. Despite malaria being endemic in over 95 % of the country, different regions of the country experience varying transmission intensities, some of which have been historically among the highest in the world [3].

Uganda is a relatively humid equatorial country although local differences in rainfall and temperature are caused by the topography, prevailing winds, and water bodies [4]. The yearly average rainfall ranges from 800 to 1500 mm, generally falling in two seasons in the south (March–May and September–November), and in one season in the north (April–October). Conversely, temperatures vary mainly with altitude and changes little from season to season. Uganda has a relatively high altitude, 1300–1500 m above sea level, and a mean annual temperature that ranges from 16 °C in the southwest to 30 °C in the northeast and 25 °C in rest of the country [5]. Vegetation is varied, with tropical rain forests in the south, wooded savanna in central Uganda and semi-desert conditions in the north [6].

Environmental factors play a major role in transmission of malaria given their relationship to both the development and survival of the mosquito and the parasite. The incubation period for malaria parasites within the mosquito is largely temperature driven, and temperature also influences the frequency of blood feeding, the rate of larval development and the survival capacity of the larvae and adult mosquito, making temperature a major determinant of malaria risk [7, 8]. At the population level, the role of temperature in the spread of malaria was examined by Mordecai and colleagues [9], using observational data from the 1900s. They demonstrated that malaria transmission peaks at 25 °C and the spread decreases dramatically at temperatures above 28 °C. Siraj et al. [10] examined malaria spatial distribution with inter-annual variability of temperature in Colombia and Ethiopia, and found that warmer years were accompanied by a higher incidence of the disease.

Precipitation results in breeding sites for the aquatic stages of the mosquito’s life cycle although severe rainfall events may wash out any mosquito larvae in these pools or aquatic environments, consequently decreasing mosquito and parasite populations [11]. Several studies have found associations between changes in malaria incidence to patterns of rainfall. For instance, a study from Sri Lanka showed strong correlations between malaria cases and rainfall with lags of 0–3 months between a rainfall event and a corresponding increase in malaria cases [12], while a study in South Africa linked inter-annual differences in malaria to rainfall [13].

In addition to temperature and rainfall, malaria incidence has also been associated with vegetation. Vegetation provides an outdoor resting refuge, serves as a food source [14] and its association with malaria incidence is well documented [15–17]. High vegetation density has been associated with higher areas of malaria transmission although conversely, low vegetation density due to intensive land use has also been associated with areas of elevated malaria transmission, given the high human population density [18].

Correlations between rainfall, temperature and vegetation with malaria morbidity have long been established. There is still, however, limited knowledge on variation of the temporal relationship (lag time) between these environmental factors with malaria morbidity resulting from changes in transmission setting. This study assessed the temporal relationship between rainfall, temperature, and vegetation with malaria morbidity across three different transmission settings in Uganda.

## Methods

### Data

The study used laboratory confirmed malaria data collected at three outpatient health facilities located at varying transmission settings in Uganda: Kamwezi [low transmission, entomological inoculation rate (EIR) < 1], Kasambya (medium transmission, EIR = 3), and Nagongera (high transmission, EIR = 562) [19]. These sites are supported to produce high quality malaria surveillance data based on laboratory confirmed cases. Weekly data from January 2010 through May 2013 was used in the study and restricted to each health facility’s catchment population [20]. Malaria morbidity was estimated using two indicators: number of malaria cases defined as total number of patients testing positive for malaria, and test positivity rate (TPR) defined as the number of patients testing positive divided by the total number of patients tested.

The study used environmental data for the catchment areas of each health facility. Data included total rainfall, average daytime temperature, and enhanced vegetation index (EVI). Satellite-based daily rainfall estimates were obtained from the Tropical Rainfall Measuring Mission (TRMM). The daily TRMM product (3B42) was extracted from NASA Goddard Earth Sciences Data and Information Services Center and it was at a spatial resolution of 0.25° × 0.25°. Temperature data was obtained from satellite estimates of land surface temperature (LST) acquired from the moderate resolution imaging spectro-radiometer (MODIS) instruments. The MODIS Terra LST product (MODI1A2) is an eight-day composite image with a 1-km spatial resolution. Mean 8-day LST (°C) were computed for each site for the daytime (10:30 a.m.) LST estimates. Vegetation data, the enhanced vegetation index (EVI), was also obtained from MODIS (MOD13A1) using 16-day composite images at a 0.5 × 0.5 km resolution. Total rainfall was the cumulative total of rainfall over a 1-week period. EVI and daytime temperature values were interpolated to a weekly temporal resolution, given the different temporal frequencies. A linear spline was used to interpolate EVI and a quadratic spline to interpolate temperature measures. All polygons were projected using the Universal Transverse Mercator System; Zone 35 North (UTM35N).

### Statistical analysis

Temporal relationships between rainfall, temperature and vegetation with malaria morbidity were examined using cross-correlation analysis with pre-whitening. Cross-correlations between each of the input series (rainfall, temperature, EVI) and the response series (malaria case counts and TPR) were analysed to detect statistically significant time lag(s) of the input series that preceded the response series with in a maximum lag of 26 weeks. Cross correlation analyses with pre-whitening involves fitting an auto regressive moving average (ARIMA) model to the input series so that the residuals are “white noise” exhibiting a random variation [21]. The same ARIMA model is then applied to the response series. Pre-whitening is used to minimize the effect of spurious correlations between the series. The residuals from each series are subsequently used to estimate the cross-correlations between the predictor and response series. To obtain pre-whitened series, various ARIMA models were applied to the rainfall, temperature and vegetation series. For each series, the ARIMA model with the lowest Akaike’s information criterion (AIC) was selected. Analysis was conducted using both SAS and STATA statistical packages. Time lags corresponding to significant cross correlations between the different environmental factors and malaria morbidity indicators were identified.

## Results

Nagongera received the most rain (30.6 mm) and experienced, on average, the highest daytime temperatures (29.8 °C) per week (Table 1). Enhanced vegetation index was similar across the three sites and these series contained the most distinct seasonality, especially for the low transmission site (Kamwezi). Contrary to known transmission intensity, weekly TPR and number of malaria cases were highest at Kasambya (medium transmission setting), followed by Nagongera (high transmission setting) and Kamwezi (low transmission setting) in the study period.

**Table 1:** Data summary

Measurement	Kamwezi (low transmission)	Kasambya (medium transmission)	Nagongera (high transmission)
Total attendance	80,808	60,248	70,484
Number suspected to have malaria	24,487	29,128	32,944
Number tested for malaria	36,167	45,134	46,901
Number of confirmed malaria	14,001	17,509	15,890
Weekly TPR	30.7 % (3.0–63.7 %)	37.0 % (12.9–83.0 %)	33.7 % (8.9–57.7 %)
Weekly case count	79 (0–533)	98 (3–513)	89 (18–223)
Total rainfall	19.1 (0–113.8)	19.0 (0–113.4)	30.6 (0–107.8)
Vegetation	0.4 (0.2–0.6)	0.5 (0.2–0.6)	0.4 (0.2–0.5)
Daytime temperature	27.6 (19.9–35.8)	27.4 (20.4–37.9)	29.8 (22.0–41.6)

The largest cross-correlation coefficient for each site was 0.27 for Kamwezi (rainfall and cases), 0.21 for Kasambya (vegetation and TPR), and −0.27 for Nagongera (daytime temperature and TPR). The study showed that temporal associations between environmental factors (rainfall, temperature and vegetation) with malaria morbidity (number of malaria cases and TPR) varied by transmission setting (Table 2). Additionally, the results differed when case counts or TPR was used. Longer time lags were observed in the relationship between rainfall and number of malaria cases at Kamwezi and Kasambya, 11–12 weeks, compared to Nagongera that showed a lag of 3 weeks. Examination of temporal relationship between rainfall and TPR showed longest time lags (23 weeks) at Kamwezi. For temperature and malaria morbidity, similar time lags (7 weeks) were observed at Kasambya and Nagongera regardless of malaria morbidity metric used. These were shorter compared to those seen at Kamwezi between temperature and TPR (25 weeks). Temporal analysis of vegetation with malaria morbidity revealed longer lags at Kasambya compared to those observed at the other two sites.

**Table 2:** Lags with their corresponding cross-correlations (statistically significant) between environmental factors and malaria

	Kamwezi (low transmission)		Kasambya (medium transmission)		Nagongera (high transmission)	
	Counts	TPR	Counts	TPR	Counts	TPR
Lags						
Rainfall	11	23	12	7	3*	7
Temperature	5	25	7	7	7*	7*
Vegetation	3	0	23	6*	3	–
Cross-correlations						
Rainfall	0.27	0.16	−0.16	0.19	0.24	0.24
Temperature	−0.15	0.15	−0.14	−0.19	0.25	−0.27
Vegetation	0.15	−0.17	−0.17	0.21	−0.15	–

* If multiple lags were significant, the lag with the highest correlation coefficient was chosen

– indicates there were no significant lags from 0 to 26 weeks

Negative relationships were observed between rainfall, temperature and vegetation with malaria case counts at Kasambya, showing that an increase in any of these input series resulted in a decrease in malaria case counts and vice versa. Similarly, negative relationships were observed between temperature and case counts at Kamwezi, temperature and TPR at Kasambya and Nagongera, between vegetation and TPR at Kamwezi and also with vegetation and case counts at Kasambya and Nagongera. The magnitude of the cross correlations between environmental factors and malaria morbidity were however comparable across sites.

The final ARIMA models were (p = 11, d = 1, q = 1), (p = 0, d = 1, q = 32) and (p = 16, d = 1, q = 1) for rainfall, daytime temperature and vegetation, respectively for Kamwezi, (p = 1, d = 1, q = 1), (p = 9, d = 1, q = 2) and (p = 4, d = 1, q = 1) for Kasambya, and (p = 9, d = 1, q = 1), (p = 12, d = 1, q = 1), (p = 18, d = 1, q = 1) for Nagongera.

## Discussion

The present study suggests that temporal associations between environmental factors with malaria morbidity vary by transmission setting in Uganda. This is the first study to examine temporal relationships between environmental factors and malaria morbidity across transmission settings in Uganda. Overall, time lags obtained in this study are consistent with those showed in other studies that have examined these associations. In Ethiopia, rainfall was positively associated with malaria cases at five out of the twelve sites with a time lag of 4–12 weeks [22] and in Rwanda, which borders Uganda in the southwest, rainfall was significantly associated with malaria incidence at 8 and 12 weeks in an area of unstable transmission [23]. The significant lags as determined by our study for malaria cases and rainfall ranged from 3–12 weeks.

This range falls within estimated time periods for the life cycle development processes of the vector and the parasite inside the vector and the host. Once an egg in laid on the surface of water, the mosquito will take, on average, 3 weeks to mature and seek a blood meal [24]. Increased flooding or rainfall could also increase the abundance of mosquitoes, given that there will be more breeding sites and increased humidity affecting mosquito longevity and parasite development [25]. Conversely, increases in severe rainfall events may wash out any mosquito larvae in these pools or aquatic environments, consequently decreasing mosquito and parasite populations [11].

This study observed significant associations between daytime temperature and malaria cases from 5 to 7 weeks. On the contrary, a study in Kenya found that temperature was associated with the greatest transmission risk of malaria 4 months prior to the peak of an epidemic [8]. More recently however, significant associations were seen at lags starting from 7 and 13 weeks in Kenya [26]. In the highlands of Burundi, a lag of 4 weeks was significant between temperature and malaria [27], while lags of 4 and 8 weeks were significant for an area of unstable malaria transmission in Rwanda [23]. Temperature has been documented to affect survival and growth of both the vector and the parasite in the vector [28–30]. Increasing temperatures will increase the speed of development of the parasite and mosquito, which in turn influences the survival of the parasite and mosquito [28]. The parasite matures in approximately one and half weeks within the mosquito at optimal temperatures while maturity in the human host takes approximately a week [24]. It is thought that temperatures of 30–32 °C are optimal for parasite and mosquito in spreading malaria [29, 30]. Therefore, if temperatures exceed 33 °C, the transmission of malaria may decrease.

In terms of vegetation and malaria cases, the present study identified significant lags of 3–23 weeks while in Burundi and Ethiopia, 8 and 12 weeks were significantly correlated to vegetation and malaria [27, 31]. As demonstrated in previous studies, malaria transmission and mosquito abundance can be influenced by vegetation cover through provision of an outdoor resting place for the vector [14].

### Limitations of the study

This study did not explore other predictors of malaria such as land use and existing control interventions, which may be important considerations for an early warning system. Additionally, the study team could not know if the observed cases were incident or recrudescent. Inclusion of recrudescent cases in the outcome series would weaken the correlation to environmental covariates [32], which have a stronger relationship with incident cases. Finally, this study did not include entomological data due to unavailability, which would provide a clearer understanding of the link between rainfall and malaria through the examination of rainfall and mosquito breeding and survival [12].

## Conclusions

This study showed that temporal associations between environmental factors with malaria morbidity vary by the three transmission settings in Uganda included in this study. The magnitude of the cross-correlation coefficients ranged from −0.27 to 0.27 with the significant lags between malaria cases and rainfall ranging from 3 to 12 weeks. For daytime temperature and malaria cases, the significant lags ranged from 5 to 7 weeks and for vegetation and malaria cases, 3–23 weeks. Results from this study highlight the need to incorporate local climatic factors when developing early warning systems for malaria control. Consideration of the variability in lags of climatic factors and malaria morbidity between transmission settings will result in development of more accurate early warning systems.

## Abbreviations

AIC: Akaike’s information criterion
ACF: autocorrelation function
AR: autoregressive
ARIMA: autoregressive integrated moving average
EIR: effective inoculation rate
EVI: enhanced vegetation index
LST: land surface temperature
MODIS: moderate resolution imaging spectro-radiometer
NASA: National Aeronautics and Space Administration
PACF: partial autocorrelation function
TPR: test positivity rate
TRMM: Tropical Rainfall Measuring Mission
UMSP: Uganda Malaria Surveillance Project
UTM35N: Universal Transverse Mercator System Zone 35 North
